# A Martyr to Duty

**Published:** 1902-01-04

**Authors:** 


### A MARTYR TO DUTY.

A subscription is being raised by Dr. William Carter, of Liverpool, on behalf of the widow and eight young children of the late Dr. William Smyth, who died as the direct result of his attending upon the inhabitants of the island of Aranmore. He was medical officer to a dispensary district in Donegal, which includes that island, and when an epidemic of typhus broke out in this remote and miserable spot, the neighbouring people refused all help, and Dr. Smyth fought the fever absolutely single-handed. Every day he rowed his boat across the stormy waters of the sound, a distance of four miles, and worked for hours in the cottages of the sick, who lay sometimes three or four in a bed. When at last he persuaded them to be removed to the mainland for better treatment, no help could be obtained, and no one would even lend a boat for the purpose. Fortunately for these miserable islanders Dr. M'Carthy, medical inspector to the Local Government Board, arrived, and these two medical men, between them, managed to bring the typhus patients down to the beach, to embark them in a boat, and to row them to the mainland, where now they are being attended to. But Dr. William Smyth caught the fever, and died a victim to duty. It will be hard indeed if something cannot be done to save from poverty the widow and the children of one who worked so courageously to help the poor.

